# Profiling Gut Microbiome and Metabolites Aids Diagnosis and Prediction of Progression in Mild Cognitive Impairment

**DOI:** 10.1002/mco2.71045

**Published:** 2026-10-06

**Authors:** Birong Zhang, Richard Brown, Antony Bayer, Joanna Waring, B. Paul Morgan, Julian R. Marchesi, You Zhou

**Affiliations:** ^1^ Division of Infection and Immunity School of Medicine Cardiff University Cardiff Wales UK; ^2^ UK Dementia Research Institute Cardiff School of Medicine Cardiff University Cardiff Wales UK; ^3^ Memory Team Cardiff and Vale University Health Board Routledge Academic Centre University Hospital Llandough Penarth UK; ^4^ Division of Digestive Diseases Department of Metabolism Digestion and Reproduction Faculty of Medicine Imperial College London London UK

## Abstract

Accumulating evidence suggests a link between the gut microbiota and metabolome and mild cognitive impairment (MCI); however, it remains largely unknown whether these parameters can aid MCI diagnosis and predict disease progression. We comprehensively characterized gut microbiota and metabolomic profiles in fecal samples from 35 individuals, including 16 spousal pairs in which one partner had MCI. Compared with controls, MCI cases exhibited significantly elevated levels of *Izemoplasmatales* spp. in the fecal microbiome and proline in the metabolome, along with decreased levels of *Ruminococcus* spp., *Lachnospira* spp., and fumaric acid. Among MCI cases, rapid progressors showed significantly increased *Bilophila* spp. in the microbiome, accompanied by higher arginine, aspartic acid, and hypoxanthine in the metabolome, and reduced *Coprococcus* spp., compared with slow progressors. Machine learning models integrating microbiota and metabolite features identified marker sets that accurately distinguished MCI cases from matched cognitively healthy controls (AUC: 0.944) and predicted rapid disease progression, with the integrated model outperforming single‐modality models for diagnosis. These findings identify distinct gut microbiota and metabolite signatures associated with MCI, highlighting their potential as candidate biomarkers for early diagnosis and risk stratification, thereby addressing the unmet need for simple tests for early detection.

## Graphical abstract

Fecal samples were collected and subjected to high‐throughput metabolomics and microbiome sequencing. Representative markers were identified for distinguishing mild cognitive impairment (MCI) cases from controls and predicting progression of cognitive decline in MCI over a year.

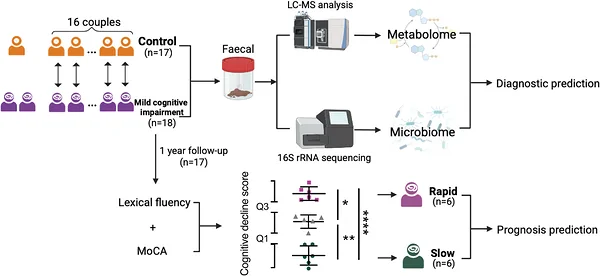

## 1 Background

Mild cognitive impairment (MCI) describes measurable cognitive decline exceeding age‐expected norms without compromising daily living activities or meeting dementia criteria. Affecting approximately 23.7% of the geriatric population globally, MCI presents a significant clinical concern due to its variable trajectory: individuals with MCI progress to Alzheimer's disease (AD) at rates of 10%–15% annually, representing a five‐toseven‐fold increase compared to cognitively normal older adults who convert at 1%–2% per year [1, 2]. This unpredictable trajectory challenges clinicians, patients, and families in making informed decisions about care planning and interventions. Accurately diagnosing and predicting which individuals will experience rapid decline remains an unmet clinical need.

Current MCI diagnosis relies on neuropsychological testing, clinical judgment, neuroimaging, and biomarkers, each with significant limitations [3]. Cognitive assessments are time consuming (1–3 h) and susceptible to confounders such as education level, language, and cultural background. Structural magnetic resonance imaging shows poor early‐stage sensitivity, while positron emission tomography imaging and cerebrospinal fluid biomarkers face accessibility barriers due to cost and invasiveness [4]. Emerging blood biomarkers targeting amyloid and tau pathologies predict MCI‐to‐AD conversion but reflect only specific pathological pathways, failing to capture the multifactorial mechanisms underlying MCI heterogeneity. Clinical scales, including the Clinical Dementia Rating and Mini‐Mental State Examination, suffer from subjectivity and ceiling effects [5]. These constraints necessitate accessible, scalable biomarkers for comprehensive MCI risk stratification.

The gut–brain axis has emerged as a promising framework in understanding cognitive impairment, with bidirectional communication between intestinal microbiota and brain function now firmly established [6]. Gut microbiomes modulate cognition through multiple mechanisms: production of neuroactive metabolites such as short‐chain fatty acids (SCFAs) and neurotransmitter precursors, regulation of blood–brain barrier integrity, modulation of neuroinflammation, and vagal nerve signaling [7, 8, 9, 10]. MCI patients exhibit dysbiosis in Firmicutes, Bacteroidetes, and Actinobacteria [11, 12, 13]. Beyond bacterial composition, microbial‐derived metabolites (SCFAs, bile acids, tryptophan derivatives, and polyamines) traverse the blood–brain barrier to directly modulate neuronal function, synaptic plasticity, and protein aggregation [14, 15, 16]. Metabolomic profiling reveals disruptions in amino acid metabolism, energy pathways, and oxidative stress, indicating metabolic dysfunction both reflects and drives cognitive decline [17, 18, 19].

Despite these insights, critical limitations hinder clinical translation. First, findings show striking inconsistency in bacterial taxa and metabolic signatures across cohorts [20, 21, 22]. Second, integrated microbiome‐metabolome analyses remain relatively scarce. Third, many studies employ cross‐sectional designs comparing MCI patients and controls at single time points [23, 24]. While such designs identify associations, they cannot predict rates of future decline trajectories. Longitudinal studies examining whether baseline gut and metabolic profiles jointly predict subsequent cognitive progression, which is crucial for prognosis and guiding therapeutic intervention, are rare. These gaps limit the translation of gut–brain axis discoveries into clinical risk stratification tools in MCI.

We conducted a longitudinal multi‐omics study integrating gut microbiome profiling, metabolomics, and machine learning to identify diagnostic and prognostic signatures in MCI. We characterized fecal samples from 35 individuals (18 MCI, 17 cognitively healthy controls), including 16 cohabiting spouse pairs to minimize environmental confounding. Crucially, 17 MCI patients underwent 12‐month cognitive follow‐up, enabling classification as rapid or slow progressors. This design tested whether baseline gut microbiome and metabolome signatures predicted subsequent cognitive decline, an essential requirement for prognostic biomarkers. We aimed to (1) identify gut microbiome–metabolome signatures that distinguish MCI from healthy controls using a cross‐sectional analysis, (2) predict the rate of cognitive decline in individuals with MCI using baseline gut microbiome–metabolome profiles and longitudinal follow‐up, and (3) evaluate whether integrated analysis outperforms single‐modality approaches. We hypothesized that baseline microbial and metabolic features would be associated with future cognitive trajectory, supporting their potential as accessible tools for MCI risk stratification.

## 2 Results

### 2.1 Study Participants and Sample Overview

A total of 35 older adults were enrolled and provided fecal samples for analysis: 18 with a diagnosis of MCI and 17 cognitively healthy controls, comprising 16 co‐habiting couples (Figure 1). The two groups were well matched for age (MCI: 75.7 ± 5.3 years; controls: 74.5 ± 4.5 years; *p* = 0.477), sex (56% vs. 53% male; *p* = 0.877), ethnicity (White ethnicity; *p* = 1.000; British/Welsh/Irish; MCI: 12:5:1; Controls: 12:4:1), formal education (13.2 ± 1.8 vs. 13.0 ± 1.9 years; *p* = 0.796), body mass index (BMI, 24.3 ± 3.4 vs. 26.6 ± 3.4 kg/m^2^; *p* = 0.062), alcohol consumption (*p* = 0.582), smoking status (1 [6%] vs. 0 current smokers; *p* = 1.000), and omeprazole use (2 [11%] vs. 2 [12%]; *p* = 1.000) (all *p* > 0.05), differing only in Montreal Cognitive Assessment (MoCA) scores (20.3 ± 3.7 vs. 27.4 ± 1.8; *p* = 0.006), as expected for their diagnostic status (Table 1). Of the 18 participants with MCI, one withdrew consent for follow‐up. The remaining 17 participants completed a 1‐year follow‐up assessment and were stratified by their composite cognitive decline ratio into rapid (*n* = 6) and slow (*n* = 6) decline groups for the prognostic analysis (Figure 1).

**FIGURE 1:**
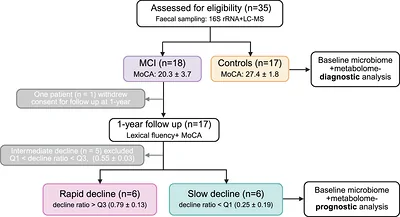
Participant screening, enrolment, and follow‐up.

**TABLE 1:** Characteristics of people with mild cognitive impairment and cognitively healthy controls.

Characteristic	MCI (*n* = 18)	Control (*n* = 17)	*p‐*value
Age, mean (SD) years	75.7 (5.3)	74.5 (4.5)	0.477^a^
Sex: Male, no. (%)	10 (56%)	9 (53%)	0.877^b^
Ethnicity: White, no. (%)	18 (100%)	17 (100%)	1.000^b^
Formal education, mean (SD) years	13.2 (1.8)	13.0 (1.9)	0.796^a^
BMI, mean (SD) kg/m^2^	24.3 (3.4)	26.6 (3.4)	0.062^a^
Current alcohol consumption, no (%)			0.582^b^
Never/rare	8 (44%)	5 (29%)	
Occasional (2–5 times/week)	7 (39%)	10 (59%)	
Most days	3 (17%)	2 (12%)	
Current smoker, no. (%)	1 (6%)	0	1.000^b^
Omeprazole medication, no. (%)	2 (11%)	2 (12%)	1.000^b^
MoCA, mean (SD)	20.3 (3.7)	27.4 (1.8)	0.006^a^

Abbreviations: BMI, body mass index; MCI, Mild cognitive impairment; MoCA, Montreal Cognitive Assessment.

^a^ Welch's *t*‐test.

^b^ Fisher's exact test.

### 2.2 Composition and Diversity of the Microbiota in Fecal Samples Differentiate MCI Cases From Controls

Fecal samples from 18 MCI cases and 17 cognitively healthy controls underwent microbiome and metabolomic analysis. A total of 1242 features were detected (combined frequency 364,175), comprising seven phyla and 138 genera. Microbiome α‐diversity (Shannon, Simpson, and inverse Simpson indices) did not differ significantly between groups, and overall β‐diversity was also similar (*p* = 0.553; Figure S1). After adjusting for household clustering using mixed‐effects models, four of the 138 identified genera differed significantly between groups: *Izemoplasmatales* (*p* = 0.0085) was enriched, while *Lachnospira* (*p* = 0.0103), *Lachnospiraceae* UCG‐008 (*p* = 0.0262), and *Ruminococcus* (*p* = 0.0492) were depleted in MCI cases compared to controls (Figure 2A, Table S1). All four genera belong to the phylum *Firmicutes*. Although *Lachnospiraceae* UCG‐008 differed significantly between groups, its apparent difference was driven predominantly by a minority of samples (80% of samples showed zero abundance, Figure 2B). It was therefore excluded from the classification model to minimize potential bias. We then developed an MCI signature comprising the remaining three genera (*Ruminococcus*, *Izemoplasmatales*, and *Lachnospira)* using five machine learning models. The artificial neural network (ANN) achieved the highest predictive performance, with an area under the receiver operating characteristic curve (AUC) of 0.972, followed by the generalized linear model (GLM, 0.944), naive Bayes (NB, 0.889), support vector machine (SVM, 0.861), and random forest (RF, 0.833 (Figure 2C, Table S2). The average AUC, accuracy, sensitivity, specificity, F1 score, and Matthews correlation coefficient (MCC) across the five models were 0.900, 0.817, 0.800, 0.833, 0.815, and 0.659, respectively, indicating that these taxa robustly distinguished MCI cases from controls (Table 2).

**FIGURE 2:**
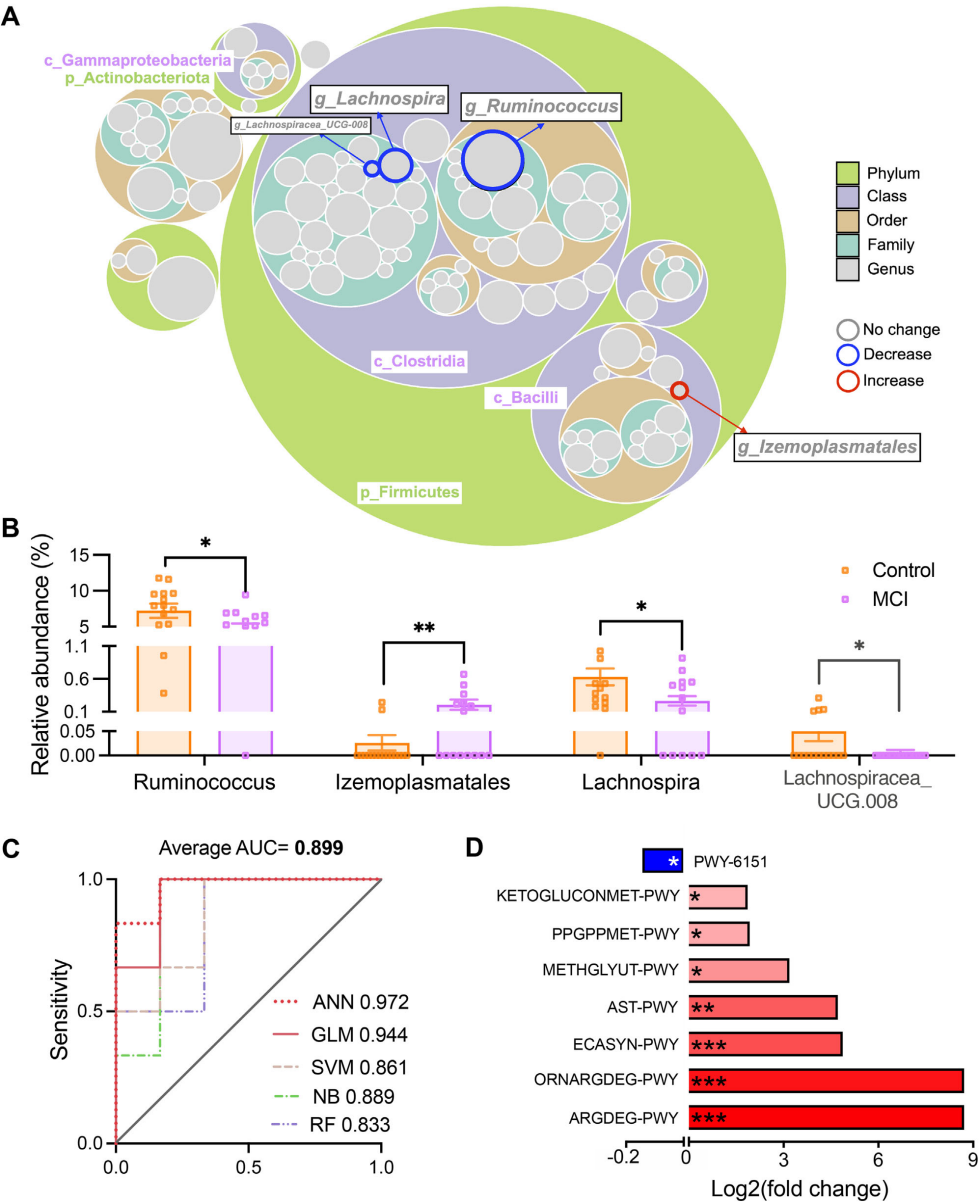
Microbiome diversity and functional changes differentiating MCI cases from controls. (A) Differential abundance of bacterial communities in fecal samples collected from MCI cases and cognitively healthy controls. Taxonomic differences are based on 16S rRNA sequences. The circles represent the phylum, class, order, family, and genus levels from outside to inside. Circles with arrows indicate taxa differing in abundance between groups (red, increased; blue, decreased, in MCI). (B) Scatter plot showing the normalized relative abundance of the four differentially abundant genera between groups (control, *n* = 17; MCI, *n* = 18; MaAsLin2: *p < 0.05). (C) ROC analysis of the microbial signature in the internal validation set. (D) Predicted functional pathways in MCI compared with controls, showing pathways enriched (red) or reduced (blue) in MCI. Pathway significance (FDR): ***p < 0.001, **p < 0.01, *p < 0.05. PWY‐6151: S‐adenosyl‐L‐methionine cycle I. ANN: artificial neural network; GLM: Generalized linear model; NB: naive Bayes; NRA: normalized relative abundance; RF: random forest; SVM: support vector machine.

**TABLE 2:** Performance metrics of models using microbial, metabolomic, and combined markers for MCI classification.

	Diagnostic model: MCI vs. control		
	Microbiome‐based (average)	Metabolite‐based (average)	Integrated model (microbiome + metabolites)
AUC	0.8999	0.8612	0.944 (0.816, 1)
Accuracy	0.8166	0.78334	0.8333 (0.5159, 0.9791)
Sensitivity	0.8000	0.79998	0.8333
Specificity	0.8333	0.76668	0.8333
F1	0.8147	0.77382	0.8333
MCC	0.6590	0.5747	0.6667

*Note*: The AUC and accuracy values represent averages across five models: artificial neural network, generalized linear model, support vector machine, naive Bayes, and random forest. For the 95% confidence intervals of these values for each individual model, please refer to Table S2.

To predict the functional capacity of the gut communities, we applied PICRUSt2; comparison between MCI cases and controls (paired Wilcoxon signed‐rank tests, accounting for the cohabiting‐pair design) identified eight differentially regulated pathways (Figure 2D). The MCI microbiome showed enrichment of L‐arginine and L‐ornithine degradation (ARGDEG‐PWY: FC = 8.714, FDR = 0.0003; AST‐PWY: FC = 4.718, FDR = 0.0017; ORNARGDEG‐PWY: FC = 8.713, FDR = 0.0003), activation of the methylglyoxal degradation super‐pathway (METHGLYUT‐PWY: FC = 3.184, FDR = 0.046), indicative of increased oxidative stress [25], enrichment of immunogenic LPS sugar components (ECASYN‐PWY: FC = 4.871, FDR = 4.57 × 10^−^^6^), and increased metabolic‐regulator (PPGPPMET‐PWY: FC = 1.859, FDR = 0.047) and ketogluconate metabolism pathways (KETOGLUCONMET‐PWY: FC = 1.927, FDR = 0.048). Conversely, S‐adenosyl‐L‐methionine biosynthesis was reduced in MCI (PWY‐6151: FC = −0.144, FDR = 0.048).

We used an independent cohort comprising samples from MCI patients (*n* = 30) and cognitively healthy controls (*n* = 30) to evaluate the diagnostic performance of the microbial biomarkers identified for MCI [20]. Consistent with our primary cohort, *Ruminococcus* was higher in the MCI group (*p* = 0.017; Figure S2A) and *Lachnospira* was lower (*p* = 0.011; Figure S2B) relative to controls. As *Izemoplasmatales* was absent from this external cohort, a signature based on the remaining two markers (*Ruminococcus* and *Lachnospira*) distinguished MCI from controls with an AUC of 0.825 (95% CI 0.605–1.0) (Figure S2C, Table S2). These findings demonstrate that the fecal microbial signature retains discriminatory ability in an independent cohort, supporting its potential utility for MCI diagnosis.

### 2.3 Fecal Metabolic Profiles Differentiate MCI Cases From Controls

The changes in microbiome composition and predicted functional led us to analyze the fecal metabolome. Liquid chromatography–mass spectrometry (LC‐MS) analyses identified a total of 5843 compounds in fecal samples, of which 119 were confidently annotated based on their mass, MS/MS spectrum, and retention time. Principal component analysis (PCA) of these annotated metabolites showed separation between MCI cases and controls (Figure 3A). After adjusting for household clustering using linear mixed‐effects models, proline (estimate = 0.0158, *p* = 0.0104) and fumaric acid (estimate = −0.0002, *p* = 0.0198) differed between groups (Figure 3B, Table S3). Proline was higher in MCI cases, while fumaric acid was lower (Figure 3C,D). Consistent directional changes were seen among the 16 cohabiting pairs, with proline increased in cases in 12 of 16 pairs and fumaric acid decreased in 11 of 16 pairs (*p* < 0.05, Figure 3C,D). We then assessed whether measurements of proline and fumaric acid adequately distinguished MCI cases from controls in five independent machine learning models. Across five machine‐learning algorithms, the AUCs ranged from 0.81 to 0.92; averaged across models, the AUC, accuracy, sensitivity, specificity, F1 score, and MCC were 0.861, 0.783, 0.800, 0.767, 0.774, and 0.575, respectively, indicating that these two metabolites contributed to discrimination between MCI cases and controls in the models (Figure 3E, Table 2, Table S2).

**FIGURE 3:**
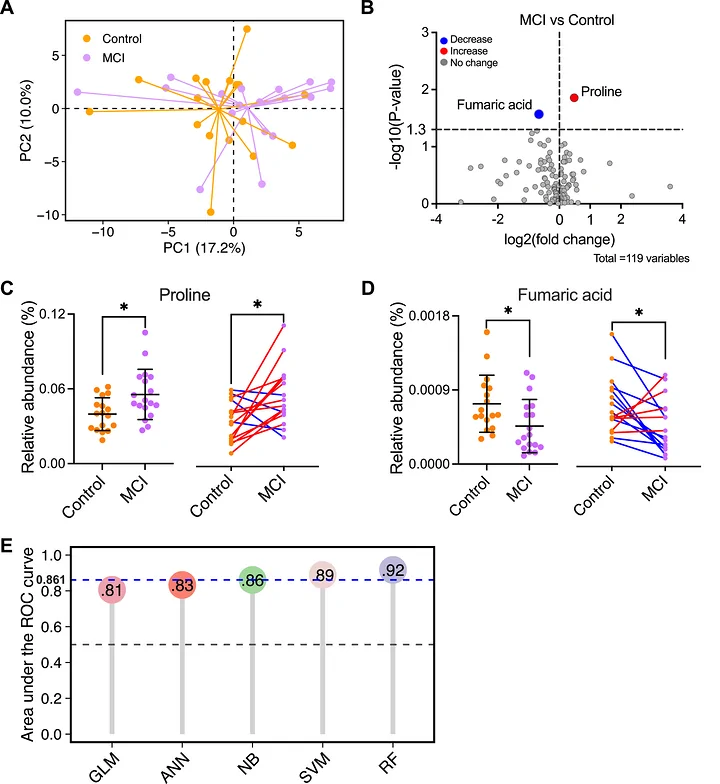
Fecal metabolic profile changes differentiating MCI cases from controls. (A) Principal component analysis of the annotated fecal metabolome in MCI cases versus cognitively healthy controls. (B) Volcano plot illustrates the log2 fold changes and corresponding *p*‐values derived from metabolomic difference analysis between MCI and controls. (C and D) Relative abundance of *proline* (C) and *fumaric acid* (D) in MCI and control groups; left panels show group comparisons (linear mixed‐effects models with household pair as a random effect), right panels show within‐pair changes for the 16 cohabiting couples (paired Wilcoxon signed‐rank test, *p < 0.05). (E) Lollipop plot showing the performance of five classification models on the internal random validation set.

### 2.4 Microbe‐Metabolite Network Patterns Differentiate MCI Cases From Controls

To investigate the interplay between gut microbiota and metabolites, we assessed correlations across all bacteria and metabolites within the MCI and control groups, uncovering 1001 correlations (*p* < 0.05). Of these, 42 involved the diagnostic markers—three genera (*Ruminococcus* spp., *Izemoplasmatales* spp., and *Lachnospira* spp.) and two metabolites (proline and fumaric acid) (Figure 4A, Table S4). In separate within‐group analyses, several microbe–metabolite relationships differed in direction between groups. For example, among the diagnostic markers, fumaric acid and *Lachnospira* were positively correlated in the MCI group but negatively correlated in controls (Figure 4B). A similar reversal was observed for arginine and *Bifidobacterium*, which were positively correlated in controls but negatively correlated in MCI (Figure 4B).

**FIGURE 4:**
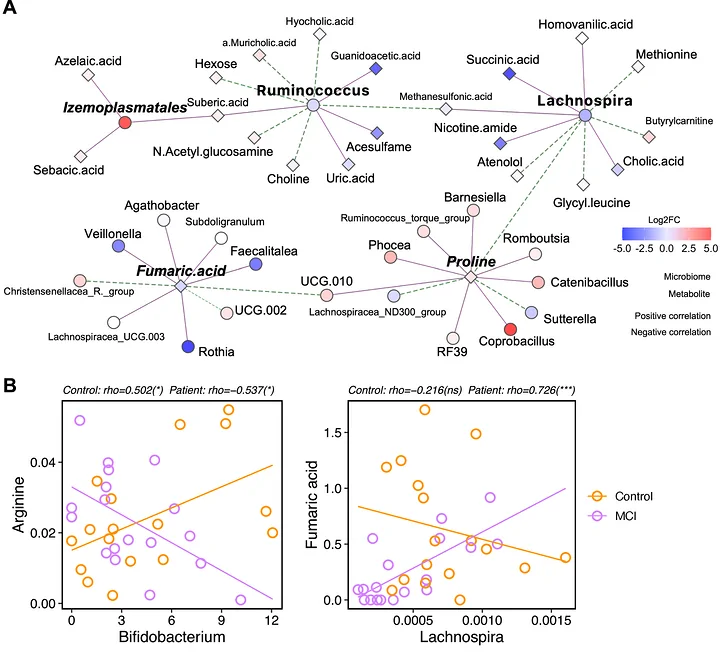
Co‐occurrence networks between microbiome genera and metabolites in MCI cases and controls. (A) Spearman correlation network showing co‐occurrence relationships between genus‐level microbes and metabolites, highlighting the five diagnostic markers (*p* < 0.05). Node shape denotes feature type (circle, microbe; diamond, metabolite), and node color indicates the log2 fold change (blue to red). Green dashed lines represent negative correlations and purple solid lines positive correlations, scaled from −1 to 1. (B) Scatter plots showing microbe–metabolite pairs whose correlation direction differed between the MCI (purple) and control (orange) groups.

### 2.5 Combining Gut Microbiota and Metabolome Strengthens Diagnostic Value for MCI

To test whether integrating microbiota and metabolites enhanced the diagnostic accuracy for MCI, SHapley Additive exPlanations (SHAP) values were used as a measure of the contribution of each variable to the outcome prediction. Each of the five markers contributed to separating MCI cases from controls (Figure 5A). *Ruminococcus* made the largest contribution (30.87% of the model output), followed by *fumaric acid* (24.54%), *proline* (16.48%), *Lachnospira* (15.35%), and *Izemoplasmatales* (12.76%) (Figure 5B). Integrating all five markers into a combined model yielded an AUC of 0.944 (95% CI 0.861–1.0) (Figure 5C, Table 2, Table S2), higher than the microbiome‐only (0.899) or metabolome‐only (0.861) models. Decision curve analysis showed that the combined model provided greater net benefit than single‐omic model across the threshold‐probability range of 0.1–1.0 (Figure 5D). Together, these results indicate that combining microbiome and metabolome markers improves diagnostic discrimination for MCI.

**FIGURE 5:**
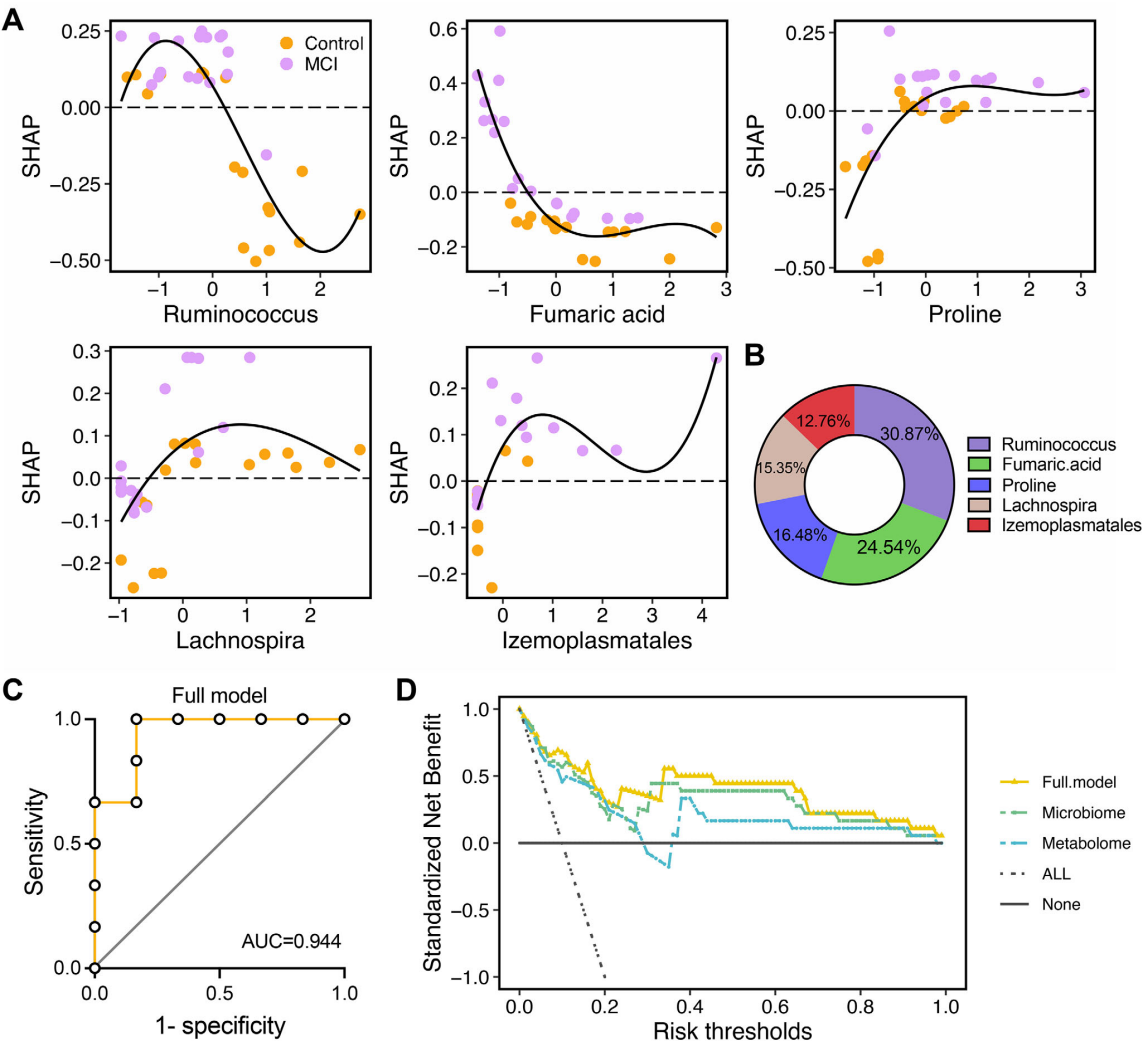
Combined effects of the microbiome and metabolome in MCI diagnosis. (A) SHAP summary plot showing the impact of the five features on the diagnostic model output. Each dot represents a participant (orange, controls: *n* = 17; purple, MCI cases: *n* = 18); the x‐axis shows the normalized abundance of each feature and the y‐axis the SHAP value. (B) Contribution of each selected feature to the classification model. (C) Performance of the combined metabolome–microbiome diagnostic model (ANN). (D) Decision curve analysis of the diagnostic models. Full model (yellow solid line): combined metabolome and microbiome model; microbiome (green dashed line): microbiome‐only model; metabolome (blue dashed line): metabolome‐only model; all (gray dashed line): assumes all samples are MCI cases; none (gray solid line): assumes all samples are controls.

### 2.6 Baseline Microbiota and Metabolite Composition Predicts Progression in MCI

To investigate whether baseline profiles predicted progression, we examined fecal microbiota and metabolites in relation to cognitive decline over 1 year. MCI cases were divided into rapid and slow progressors using each participant's decline ratio. Shannon and InvSimpson indices demonstrated a reduction in microbiome α‐diversity, significant in the latter index, in the rapid cognitive decline group compared with the slow decline group (Figure 6A). The overall β‐diversity, reflecting community composition in the microbiota signatures, was comparable between the two groups (*p* = 0.10, Figure 6B). At the genus level, five genera (*Bacteroides*, *Faecalibacterium*, *Christensenellaceae R‐7 group*, *Blautia*, and *Ruminococcus*) accounted for 20.15% of the total taxonomy in the slow group and 37.21% in the rapid group (Figure 6C). Notably, mean *Bacteroides* abundance in the rapid group (17.04%) was almost double that in the slow group, *Bifidobacterium* was reduced (rapid 2.34%; slow 5.11%), and *Streptococcus* was more abundant (6.46% vs. 1.82%) (Figure 6C). Differential abundance analysis identified *Bilophila* (*p* = 0.0345) as significantly enriched and *Coprococcus* (*p* = 0.0455) as significantly reduced in the rapid group (Figure 6D). A microbiota signature based on these two genera predicted rapid progression across five machine‐learning algorithms, with an average AUC of 0.867 (accuracy, sensitivity, and specificity 0.800; F1 0.796) (Figure 6E, Table 3). To further assess the signature in an independent cross‐sectional cohort, we analyzed patients with established AD (*n* = 58) and MCI (*n* = 14) [26]. The signature based on *Coprococcus* and *Bilophila* yielded an AUC of 0.72 (95% CI: 0.506–0.934) for distinguishing AD from MCI (Figure S2D, Table S2). These findings are consistent with the potential relevance of the signature to MCI progression and warrant confirmation in future longitudinal studies.

**FIGURE 6:**
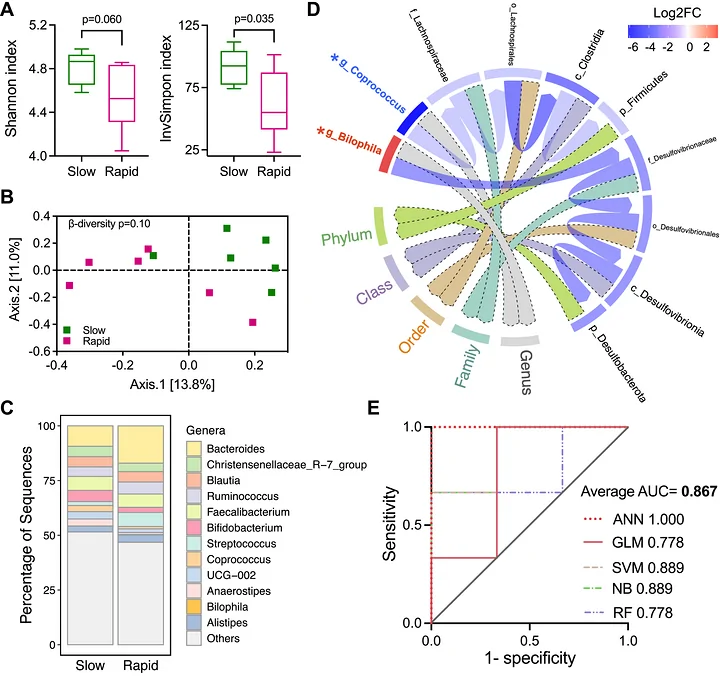
Microbiome composition in MCI cases with rapid versus slow progression. (A) α‐diversity (Shannon and inverse Simpson indices; slow, *n* = 6; rapid, *n* = 6). (B) β‐Diversity (Bray–Curtis distances). (C) Relative proportions of bacterial taxa at the genus level. (D) Differential abundance of bacterial taxa between rapid and slow progressors. The outermost layer shows the log2 fold change of each taxon; relative to the slow group, red indicates increased and blue decreased abundance in the rapid group. Internal connecting lines are color‐coded by taxonomic level. (E) Performance of the classifiers in predicting progression using the genus‐level microbial signature.

**TABLE 3:** Performance metrics of models using microbial, metabolomic, and combined markers for predicting disease progression.

	Prognostic model: Rapid vs. slow		
	Microbiome‐based model (average)	Metabolite‐based model (average)	Integrated model (microbiome + metabolites)
AUC	0.8668	0.9556	1 (1,1)
Accuracy	0.79998	0.89998	1 (0.5407,1)
Sensitivity	0.80002	1	1
Specificity	0.80002	0.80002	1
F1	0.7961	0.88	1
MCC	0.6323	0.8243	1

*Note*: The AUC and accuracy values represent averages across five models: artificial neural network, generalized linear model, support vector machine, naive Bayes, and random forest. For the 95% confidence intervals of these values for each individual model, please refer to Table S2.

Gut metabolite profiles also differed between groups, with higher relative abundance of arginine, aspartic acid, and hypoxanthine in rapid progressors (Figure 7A). Within the 12 rapid and slow progressors, these three metabolites correlated positively with the degree of cognitive decline (arginine: *r* = 0.88, aspartic acid: *r* = 0.95, hypoxanthine: *r* = 0.93; all *p* < 0.001, Figure 7B). PCA on these three metabolites separated the rapid and slow groups (Figure 7C). Across five machine‐learning algorithms, a metabolite‐based model achieved an average AUC of 0.956 (accuracy 0.900, sensitivity 1.000, specificity 0.800, F1 0.880, MCC 0.824) (Figure 7D, Table 3).

**FIGURE 7:**
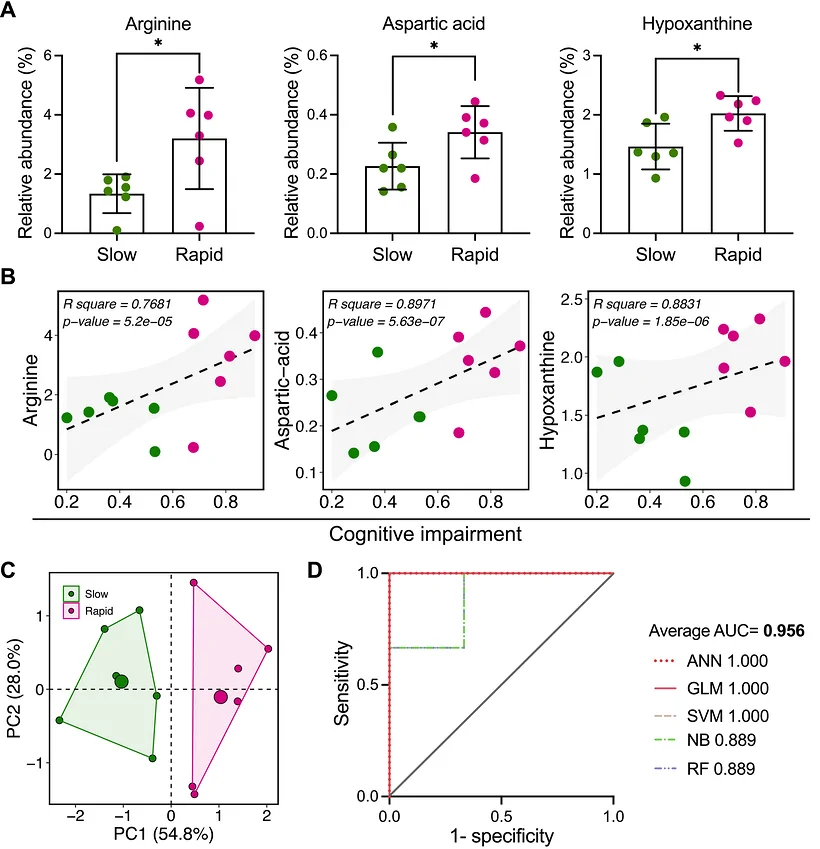
Fecal metabolite profiles in MCI cases with rapid versus slow progression. (A) Relative abundance of arginine, aspartic acid, and hypoxanthine in the rapid and slow progression groups. (B) Correlation between the rate of cognitive decline and the three metabolites (analysis restricted to the rapid and slow progressors, *n* = 12; exploratory). (C) PCA based on arginine, aspartic acid, and hypoxanthine showing separation between the progression groups. (D) ROC curves for five algorithms predicting progression; AUC values from repeated random training–validation splits are listed on the right.

Finally, to examine the combined markers, we performed SHAP analysis as for the diagnostic models. Of 724 significant correlations across all bacteria and metabolites in baseline samples, 37 correlations involved the prognostic markers identified above: bacterial genera *Bilophila*, *Coprococcus*, and metabolites arginine, aspartic acid, and hypoxanthine (Figure 8A, Table S5). The combination of just the three metabolites (arginine, aspartic acid, and hypoxanthine) contributed to 99.97% of the model output in predicting progression of cognitive impairment, rendering the contribution of bacterial genera negligible (Figure 8B). Consistent with this, decision curve analysis further demonstrated that both the full model and the metabolome‐only model significantly outperformed the microbiome‐only model (Figure 8C). In contrast, there was negligible difference in classification performance between the full model and the metabolome‐only model across the entire range of risk thresholds (Figure 8C). These findings suggest that microbial features added little incremental value once the stronger metabolite signal was included in the current dataset.

**FIGURE 8:**
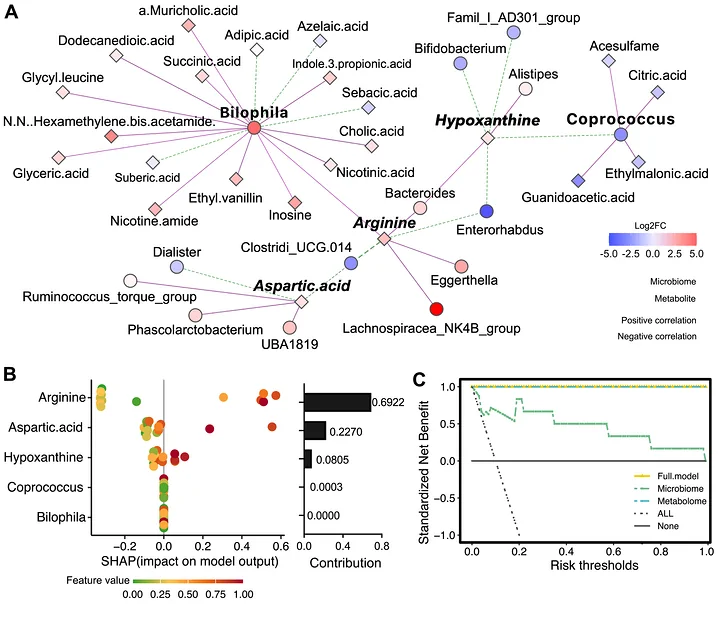
Co‐occurrence networks and feature contributions in MCI cases with rapid versus slow progression. (A) Spearman correlation network showing co‐occurrence relationships between genus‐level microbes and metabolites, with a specific emphasis on five distinct indicators (*p*‐value < 0.05). The node shape denotes the feature type (circle for microbe, diamond for metabolite), while color signifies the log2 fold change, transitioning gradually from blue to red. Dashed lines (green) represent negative correlations, whereas solid lines (purple) denote positive correlations, with colors scaling from −1 to 1. (B) Beeswarm plot (left) showing the association between features and SHAP values, with each point representing a participant (*n* = 12); point color indicates the normalized feature abundance (green to red). The bar graph (right) shows the contribution of each feature to the model prediction. (C) Decision curve analysis of the prognostic models. Full model (yellow solid line): combined metabolome and microbiome prognostic model; microbiome (green dashed line): microbiome‐only prognostic model; metabolome (blue dashed line): metabolome‐only prognostic model; all (gray dashed line): assumes all samples are rapid progressors; none (gray solid line): assumes all samples are slow progressors.

## 3 Discussion

In this study, we investigated whether fecal microbiota and metabolomic profiles could aid diagnosis and prediction of cognitive decline trajectory in MCI. We characterized gut microbiome and metabolome profiles in 35 individuals (18 MCI, 17 cognitively healthy controls, including 16 cohabiting spouse pairs) at MCI patients undergoing 12‐month prospective cognitive follow‐up. This design enabled machine‐learning models for both cross‐sectional MCI diagnosis and longitudinal prediction of cognitive decline rate. To our knowledge, this is the first study to examine whether baseline fecal microbiota and metabolome profiles can both identify MCI and track the subsequent rate of cognitive decline, potentially supporting early diagnosis and prognostic risk stratification in MCI.

Several published studies have documented alterations in the composition of gut microbiota in MCI, preclinical AD, and established AD [22, 27]; however, the variability in methods and markers employed across studies makes comparison challenging. While we took a broad approach to marker identification, several published studies have focused on specific groups of organisms or metabolites. We found significant alterations in bacterial composition in MCI cases compared with matched controls, most of whom were co‐habiting partners, selected to reduce the contribution of environmental variability. Microbiota changes associated with “caseness” were enrichment of *Izemoplasmatales* and reduction of *Lachnospira* and *Ruminococcus*; all three of the identified genera belonged to phylum *Firmicutes*, alterations of which have been reported in other studies [28, 29]. We further identified eight differentially regulated metabolic pathways linked to changes in the microbiota, including three (ARGDEG‐PWY, AST‐PWY, and ORNARGDEG‐PWY) involved in L‐arginine degradation and linked to the tricarboxylic acid (TCA) cycle, previously associated with AD [30]. Others have shown that alterations in gut microbiota are often accompanied by changes in bacterial metabolites that may impact host health and disease [31]; notably, progression of AD has been linked to metabolism of D‐aspartic acid and N‐acetyl‐L‐aspartic acid [32, 33]. Guided by these studies and the observed pathway changes, we performed an LC‐MS analysis of metabolites in the collected samples; two metabolites, *proline* (increased abundance in cases) and *fumaric acid* (reduced abundance in cases), stood out in the analysis.

We next used bioinformatic approaches, including machine learning models, to test whether the observed differences in the microbiome and/or metabolome were of diagnostic value for differentiating MCI from controls. Analysis of microbiota identified an MCI signature comprising three genera (*Ruminococcus, Izemoplasmatales*, and *Lachnospira*) that predicted MCI across five machine learning models (average AUC 0.899). A metabolite signature comprising proline and fumaric acid predicted MCI across the machine‐learning models (average AUC 0.861). A combined model including all five parameters reached an AUC of 0.944 for distinguishing MCI from controls.

Increasing evidence suggests that changes in the gut microbiota and metabolome may predict progression of complex diseases; for example, species‐level taxonomic changes in the microbiome predicted the development of type 2 diabetes, microbiome markers predicted progression of non‐alcoholic fatty liver disease 4 years later, and microbiome and metabolomic profiles predicted the progression of cardiovascular disease [33, 34, 35, 36]. We tested whether changes in gut microbiota and/or metabolome were associated with progression in MCI. Our first observation was that microbiome diversity was reduced in the group that progressed rapidly compared to slow or no progressors, supporting previous reports [28, 37]. At the genus level, *Bilophila* was increased and *Coprococcus* was reduced in rapid progressors; models including these two genera achieved good predictive performance (AUC 0.867). Three metabolites (arginine, aspartic acid, and hypoxanthine) were also elevated in rapid progressors; models incorporating these metabolites showed stronger predictive performance (AUC = 0.956). This stronger metabolite signal explains why microbial features contributed negligibly to the integrated model: the metabolite‐only model substantially outperformed the microbiome‐only model (AUC 0.956 vs. 0.867). This does not indicate an absence of microbial signal but rather reflects the greater discriminative capacity of the metabolite features in this dataset. Accordingly, SHAP attribution assigned greater importance to the more informative metabolite features, which are likely to lie closer to the cognitive phenotype and provide a more proximal functional readout than taxonomic abundance [38]. Consequently, although the microbial features were independently predictive, they contributed little additional information once metabolite features were included in the integrated model. This pattern is specific to the present dataset and should not be interpreted as indicating that metabolomic markers are inherently superior to microbial markers across diseases.

Our study has several limitations. First, the cohort size was limited, increasing the risk of overfitting and potentially inflating the apparent predictive performance of the model. Although we assessed performance across five machine‐learning algorithms using repeated random training–validation splits, such approaches cannot fully compensate for this limitation. Consequently, the reported predictive performance may overestimate the model's true generalizability to independent patient populations. The perfect performance of the integrated prognostic model should therefore be interpreted with caution rather than considered established predictive accuracy. Second, while the diagnostic signatures were validated in an independent external cohort, no independent longitudinal cohort was available for external prognostic validation. The prognostic signature was therefore examined only in a cross‐sectional AD/MCI cohort, which provides supporting rather than confirmatory evidence for its relevance to disease progression [20, 26]. These findings require confirmation in larger, independent longitudinal cohorts before any claims of clinical predictive utility can be made. Third, although the participants met the core clinical criteria for MCI, amyloid and tau pathological biomarkers were not available. The cohort was therefore not biomarker‐confirmed for underlying AD pathology, and we were unable to distinguish AD‐type MCI from other MCI subtypes. This limits the specificity and generalizability of our findings across MCI subtypes, and confirmation in biomarker‐defined cohorts remains an important direction for future research. Fourth, the microbiome analysis was based on 16S rRNA amplicon sequencing, which provides reliable taxonomic classification at the genus level but does not allow robust species‐level resolution. Future studies using higher resolution approaches such as metagenomic sequencing may help clarify species‐level associations within genera, which may exert distinct biological effects. Other limitations include the lack of data on dietary intake, a known modulator of gut microbiota composition, other lifestyle, environmental and genetic confounders (e.g., APOE genotype), and impact of ethnicity (all participants were of White ethnicity) [39, 40, 41].

In summary, we identify baseline changes in the gut microbiota and metabolome that distinguish MCI cases from controls and that are associated with the rate of cognitive decline over 1 year. These findings highlight the potential of gut microbiota‐ and metabolome‐based approaches for the early diagnosis and prognostic stratification of MCI, and raise the possibility of targeting the microbiota in disease‐modifying strategies, while underscoring the need for validation in larger independent cohorts.

## 4 Materials and Methods

### 4.1 Study Participants

All procedures were conducted in accordance with the principles of the Declaration of Helsinki and received approval from the Wales Research Ethics Committee 1 (REC # 19/WA/0338) and NHS Health Research Authority. Written informed consent was obtained from each participant upon enrolment. Participants comprised 18 individuals with MCI and 17 cognitively healthy elderly controls, including 16 co‐habiting couples in which one partner was diagnosed with MCI and the other was cognitively healthy. Cases met the core clinical criteria for MCI, and cognitive health of the controls was confirmed by a MoCA score ≥ 25 and no cognitive concerns [42]. Exclusion criteria in both groups were use of antibiotics or probiotics/prebiotics in the 4 weeks before fecal sample collection and confounding conditions such as presence of major neurological or psychiatric disorder, alcohol abuse, cancer, coeliac disease or inflammatory bowel disease, or medication that might adversely affect cognition. Amyloid and tau pathological biomarkers were not available for the participants in this study; consequently, the MCI cohort was defined on clinical grounds and was not biomarker‐confirmed for underlying AD pathology. APOE status was also not available.

At the 1‐year follow‐up assessment, cognitive function was re‐evaluated in 17 participants from the initial MCI cohort using the MoCA and lexical fluency. Cognitive decline ratios were calculated for each participant by averaging the proportional changes in MoCA and lexical fluency scores from baseline to follow‐up. These ratios were subsequently normalized to a continuous scale ranging from 0 to 1, where higher values indicated greater cognitive deterioration (Table S6). Participants were further stratified according to the quartile distribution of the composite decline ratios. Those with decline ratios above the third quartile (Q3) threshold were classified as experiencing rapid cognitive decline (*n* = 6), representing the most pronounced deterioration within the cohort. Conversely, participants with decline ratios at or below the first quartile (Q1) were categorized as having slow cognitive decline (*n* = 6), indicating relative cognitive stability over the observation period. Participants with intermediate decline ratios (Q1–Q3; *n* = 5) were not included in the between‐group prognostic comparison (*n* = 5). This extreme‐phenotype stratification approach was adopted to maximize phenotypic contrast between the comparison groups for biomarker discovery.

### 4.2 Sample Collection and Processing

Fecal samples were collected in the home setting for all 35 participants using the EasySampler stool collection kit (Fisher Scientific). Upon collection, the samples were promptly transferred to the laboratory, where they were placed in sterile tubes and stored frozen at −20°C until processed. Prior to analysis, each fecal sample was thawed at ambient temperature in a sterile environment.

### 4.3 DNA Extraction and Sequencing Procedure

For DNA extraction, an aliquot (200–400 mg wet weight) was extracted from the bulk fecal sample using a sterile spatula and transferred to a sterile, pre‐labelled microcentrifuge tube (2 mL with O‐ring screw cap). Initial processing involved homogenization using a bead‐beater (MP Fastprep‐24 5G; five cycles of 4.5 m/s for 45 s with 5 min on ice between each cycle). DNA extraction was then carried out using the Quick Faecal‐Soil microbe DNA mini kit (Zymo research D6010) following the manufacturer's protocol. DNA was extracted in a volume of 100 µL, and its yield and integrity were assessed by measuring absorbance (A260/A320 and A260/A280, respectively) on a Nanodrop spectrophotometer (ThermoFisher) and stored in low‐binding 1.5‐mL microfuge tubes.

### 4.4 16S rRNA Gene Sequencing Analysis of Fecal Samples

DNA amplicons were generated using a two‐step PCR process prior to sequencing. In the first step, the targeted V1/V2 (28F/388R) region and the overhangs of the Illumina adapters were amplified and run for 25 cycles, followed by cleaning and validation using Qiaxcel (Quiagen) and Qubit (ThermoFisher) assays. In the second step, indexes were added to each sample along with the remainder of the adapters and eight cycles were performed to facilitate indexing. The quality and quantity of the amplicons were then cleaned and validated using Tapestation (Agilent) and Qubit, and their concentrations were calculated in nM. The amplicons, normalized by quantity between samples, were then subjected to 16S rRNA sequencing on an Illumina MiSeq sequencer at the Cardiff University Genomics Center. To capture a broad diversity, 16S rRNA gene sequencing was performed using four different forward primers (28F‐YM, 28F‐Borrellia, 28FChloroflex, and 28F‐Bifdo) in a 4:1:1:1 ratio. Primer sequences are shown in Table S7.

The sequencer output, provided as deconvoluted adapter and indexer pre‐trimmed paired FASTQ files, underwent pre‐processing and subsequent taxonomy analysis and functional annotation using qiime2 on the HAWK High‐Performance Computing (HPC) cluster (https://portal.supercomputing.wales/index.php/about‐hawk/) [43]. Quality control and trimming of the raw paired‐end reads were executed using Cutadapt and DADA2, respectively [44]. To mitigate the potential bias from mixed primers during training using the q2‐feature‐classifier function, the similarity between the four primers was evaluated (78.95%), enabling the selection of an identity threshold of 0.75. Consequently, the 28F‐YM primer was selected as the primary primer, permitting matches with the other three primers. A naive Bayes classifier trained on the Silva v138 database (https://www.arb‐silva.de/documentation/release‐138/) was employed to assign taxonomies to the features, and Scikit‐learn was used to validate the classification process. The result contained 5134 features with a cumulative frequency of 476,137. Any amplicon sequence variants (ASVs) that were present in fewer than two samples or appeared less than 10 times were excluded from the analysis, yielding 1242 unique ASVs for further investigation with a median of 9112 reads. Phyloseq was used to estimate diversity within and between samples [45]. Microbiome diversity within a habitat type and dissimilarity between two communities were measured using α‐ and β‐diversity, respectively. α‐Diversity was measured using Shannon and Inverse Simpson (InvSimpson) indices and compared using the Mann–Whitney test. β‐Diversity was calculated based on Bray–Curtis distance and compared using permutational multivariate analysis of variance. The potential functions of bacterial community were predicted by PICRUSt2 [46] and were compared using paired Wilcoxon signed‐rank tests. Absolute feature abundances were converted to relative abundances by dividing each value by the total abundance within the corresponding sample. For the diagnostic analysis, to account for the non‐independence introduced by the 16 cohabiting spouse pairs, differential abundance between MCI cases and controls was assessed using mixed‐effects models implemented in MaAsLin2 [47], with group (MCI vs. Controls) as a fixed effect and household pair as a random effect; relative abundances were arcsine square‐root transformed prior to analysis. For the prognostic analysis, differential abundance between rapid and slow progressors was assessed using ZicoSeq [48].

### 4.5 Metabolomic Analysis of Fecal Samples

Metabolic analyses were performed at MS‐omics (https://www.msomics.com). Fecal samples were extracted in house using the MS‐Omics fecal extraction protocol. In brief, 100 mg feces sample was placed into a labeled 1.5‐mL microcentrifuge tube, homogenized in MilliQ water (400 µL) using a bead beater (MP Fastprep‐24 5G; 4.5 m/s for 45 s), and centrifuged (16,000 *g*, 4°C, 10 min). The supernatant was transferred into Costar SpinX filter tubes (0.22‐µm filter) and centrifuged at 15,000 *g*/4°C for 5 min. The filtrate was aliquoted and stored at −80°C before being shipped on dry ice to MS‐Omics for analysis of metabolites using a Thermo Scientific Vanquish LC coupled to Thermo Q Exactive HF MS with an electrospray ionization interface source. Peak areas were extracted using Compound Discoverer 3.1 (Thermo Scientific), and compounds were identified by retention times (compared with in‐house standards; accepted deviation of 3 ppm) with or without MS/MS spectral data. For the diagnostic analysis, metabolite data were normalized using the same procedure applied to the microbial data. Differential abundance between MCI cases and controls was assessed using linear mixed‐effects models (lmerTest package [49]), with group (MCI vs. control) as a fixed effect and household pair as a random effect to account for the 16 cohabiting spouse pairs. Paired Wilcoxon signed‐rank tests were additionally performed as supporting analyses. For the prognostic analysis, differential abundance between rapid and slow progressors was assessed using the Mann–Whitney *U* test, as these groups comprised independent MCI participants without the paired household structure of the diagnostic comparison.

### 4.6 Biomarker Identification and Machine Learning Classifier Construction

For diagnostic prediction, the 35 samples were partitioned into training and validation subsets by stratified random sampling to maintain balanced class representation, using an approximately 7:3 ratio (23 training, 12 validation). For the prognostic analysis, the 12 rapid and slow progressors remaining after exclusion of the five participants with intermediate cognitive decline ratios (see Section 4.1) were split into training (*n* = 6) and validation (*n* = 6) sets in the same manner. Classification models were built using five machine‐learning algorithms: ANN, GLM, SVM, NB, and RF. Performance was assessed across repeated random training–validation splits, with samples re‐partitioned at each iteration so that each validation set differed from previous ones. Features were selected using a predetermined nominal threshold (*p* < 0.05) in the differential abundance analyses of the microbiota and metabolome; feature selection was performed on the full dataset. For the integrated microbiota–metabolome models, feature contributions were quantified using SHAP [50], which estimate the contribution of each feature to model predictions and support identification of the most influential markers.

Model performance was evaluated by receiver operating characteristic (ROC) curve analysis, with AUC and 95% confidence interval (CI) as the primary metric. Additional metrics, including accuracy (with 95% CI), sensitivity, specificity, F1 score, and MCC, were also reported. Decision curve analysis was performed to compare models by plotting standardized net benefit against risk thresholds using the DecisionCurve package [51]. The study was reported in accordance with the Transparent Reporting of a multivariable prediction model for Individual Prognosis Or Diagnosis (TRIPOD) guideline [52], and a completed checklist was provided for peer review.

### 4.7 External Datasets and Model Performance Assessment

To examine whether the identified microbial markers generalized beyond the discovery cohort, two independent external datasets were analyzed. For the diagnostic markers, a balanced cohort comprising 30 individuals with clinically diagnosed MCI (*n* = 30) and 30 cognitively healthy controls (*n* = 30) was used [20]. For the prognostic markers, a separate cross‐sectional cohort comprising individuals with MCI (*n* = 14) and established AD (*n* = 58) was examined [26]. All external samples were fecal. Raw microbial sequencing data underwent the same quality‐control and taxonomic‐analysis procedures described in Section 4.4, ensuring methodological consistency between the discovery and external datasets. ANN models were used to assess the performance of the identified markers in these datasets, and performance was evaluated by ROC analysis, with AUC values calculated to quantify diagnostic and prognostic accuracy.

### 4.8 Statistical Analysis

All statistical analyses were performed in R 4.1.0 (https://www.r‐project.org) and GraphPad Prism 8.0. Multiple comparison correction was carried out using the Benjamini–Hochberg method. A two‐sided *p* value of less than 0.05 was considered statistically significant.

## Author Contributions

Bioinformatics and statistical analyses, interpretation of data, drafting of the manuscript, critical revision of the manuscript, and statistical analysis: Birong Zhang. Acquisition of data, statistical analyses, and drafting of the manuscript: Richard Brown. Acquisition of data, analysis and interpretation of data, and critical revision of the manuscript: Antony Bayer and Joanna Waring. Study concept and design, analysis and interpretation of data, drafting of the manuscript, critical revision of the manuscript, and study supervision: B. Paul Morgan, Julian R. Marchesi, and You Zhou. All authors have read and approved the final manuscript.

## Funding

This project was funded by the Rosetrees Trust (Project Grant to B.P.M.; Reference A2226). J.R.M. and the Division of Digestive Diseases at Imperial College London receive financial and infrastructure support from the NIHR Imperial Biomedical Research Centre (BRC) based at Imperial College London.

## Ethics Statement

All procedures were conducted in accordance with the principles of the Declaration of Helsinki and received approval from the Wales Research Ethics Committee 1 (REC # 19/WA/0338) and NHS Health Research Agency. All participants provided written informed consent upon enrolment. The external cohorts used for validation were previously published and had received ethical approval [20, 26].

## Conflicts of Interest

The authors declare no conflicts of interest.

## Supporting information




**Supplementary Table 1**: Differential abundance analysis of fecal microbial genera between MCI cases and controls using mixed‐effects models, with group (MCI vs control) as a fixed effect and household pair as a random effect.

**Supplementary Table 2**: Performance metrics of all models based on microbial, metabolomic, and combined markers for diagnosis or prognosis.

**Supplementary Table 3**: Differential abundance analysis of fecal metabolites between MCI cases and controls using linear mixed‐effects models, with group (MCI vs control) as a fixed effect and household pair as a random effect.

**Supplementary Table 4**: Correlations between genus‐level microbes and metabolites in the diagnosis of MCI.

**Supplementary Table 5**: Correlations between genus‐level microbes and metabolites in the prognosis of MCI.

**Supplementary Table 6**: Classification of MCI progression over 12 months.

**Supplementary Table 7**: Sequence of primer used for 16S rRNA sequencing.

**Supplementary Figure 1**: Comparison of microbial diversity between mild cognitive impairment (MCI) cases and controls.
**Supplementary Figure 2**: External evaluation of the microbial signatures. (A–C) Diagnostic markers in an independent cohort. Relative abundance of (A>) Ruminococcus and (B) Lachnospira in normal controls (NC) and MCI cases. (C) Diagnostic performance of the artificial neural network (ANN) model in this external cohort (AUC = 0.825). (D) Performance of the ANN model in a cross‐sectional AD/MCI cohort, distinguishing AD from MCI (AUC = 0.720).

## Data Availability

The metabolomics data are available on Figshare: https://figshare.com/s/c0503c90c55a54ad2dc5?file=55441892. Raw 16S rRNA sequencing data are available in the NCBI BioProject database under accession number PRJNA1210544: https://www.ncbi.nlm.nih.gov/bioproject/PRJNA1210544. Microbiota data from external cohorts are available at NCBI BioProject (accession number: PRJNA489760, https://www.ncbi.nlm.nih.gov/bioproject/PRJNA489760) and European Nucleotide Archive (accession number: PRJEB59009, https://www.ebi.ac.uk/ena/browser/view/PRJEB59009)
