# Supplementary material for: Profiling Gut Microbiome and Metabolites Aids Diagnosis and Prediction of Progression in Mild Cognitive Impairment
Source: MedComm (2020). 2026 Oct 6;7(10):e71045. doi: 10.1002/mco2.71045 (PMC13639709; doi:10.1002/mco2.71045)
Supplement: Supplementary file 1 [file MCO2-7-e71045-s006.pdf]

Supplementary Information

for

Profiling Gut Microbiome and Metabolites Aids Diagnosis and Prediction of Progression in Mild Cognitive Impairment

Birong Zhang[1], Richard Brown[1,2], Antony Bayer[2,3], Joanna Waring[3], B. Paul Morgan[1,2*], Julian R. Marchesi[4*] and You Zhou[1*].

[1]Division of Infection and Immunity and [2]UK Dementia Research Institute Cardiff, School of Medicine, Cardiff University, Heath Park, Cardiff CF14 4XN; [3]Memory Team, Cardiff and Vale University Health Board, Routledge Academic Centre, University Hospital Llandough, Penarth CF64 2XX, UK; [4]Division of Digestive Diseases, Department of Metabolism, Digestion and Reproduction, Faculty of Medicine, Imperial College London, London, UK.

*** Correspondence:**

B. Paul Morgan, Ph.D., UK Dementia Research Institute Cardiff and Division of Infection and Immunity, School of Medicine, Cardiff University, Cardiff, Wales CF14 4XN, United Kingdom. Email: MorganBP@cardiff.ac.uk

Julian R. Marchesi, Ph.D., Division of Digestive Diseases, Department of Metabolism, Digestion and Reproduction, Faculty of Medicine, Imperial College London, London, UK. Email: j.marchesi@imperial.ac.uk

You Zhou, Ph.D., Division of Infection and Immunity, School of Medicine, Systems Immunity Research Institute, Cardiff University, Cardiff, Wales CF14 4XN, United Kingdom. Email: zhouy58@cardiff.ac.uk

Supplementary Figure 1. Comparison of microbial diversity between Mild Cognitive Impairment (MCI) cases and controls.

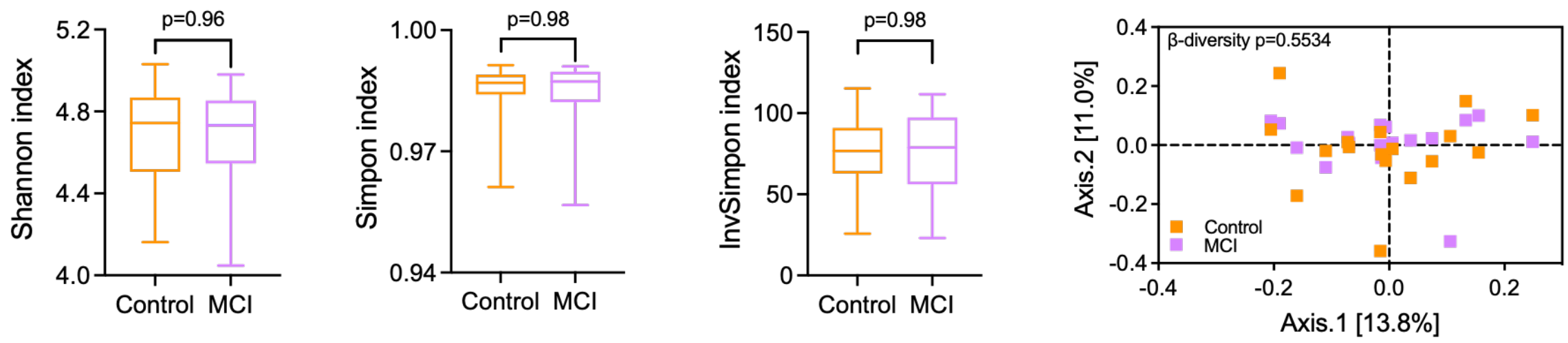

Supplementary Figure 2. External evaluation of the microbial signatures. (A–C) Diagnostic markers in an independent cohort. Relative abundance of (**A**) Ruminococcus and (**B**) Lachnospira in normal controls (NC) and MCI cases. (**C**) Diagnostic performance of the artificial neural network (ANN) model in this external cohort (AUC= 0.825). (**D**) Performance of the ANN model in a cross-sectional AD/MCI cohort, distinguishing AD from MCI (AUC = 0.720).

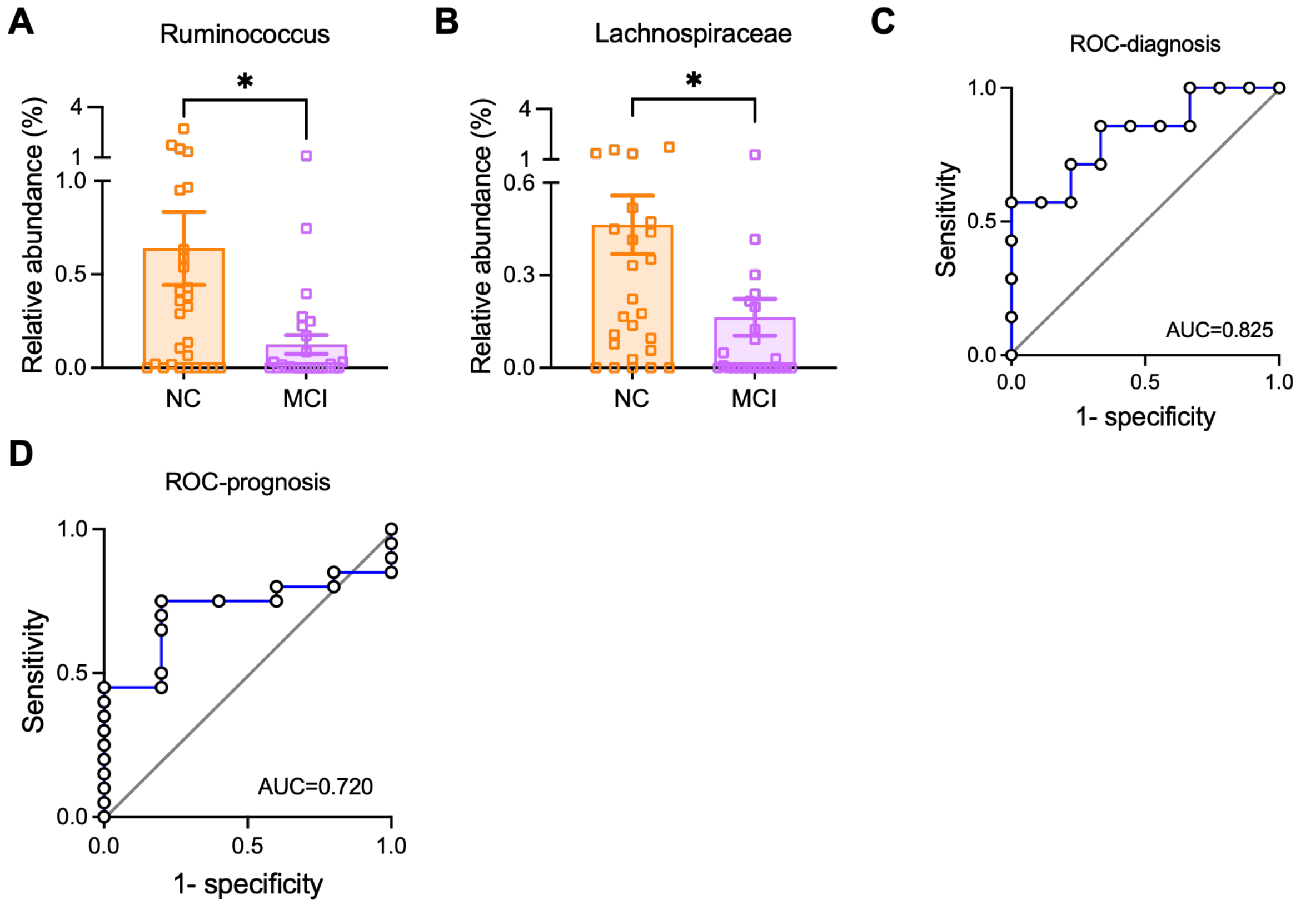

Supplementary Table 1. Differential abundance analysis of faecal microbial genera between MCI cases and controls using mixed-effects models, with group (MCI vs control) as a fixed effect and household pair as a random effect.

Supplementary table 2. Performance metrics of all models based on microbial, metabolomic, and combined markers for diagnosis or prognosis.

Supplementary Table 3. Differential abundance analysis of faecal metabolites between MCI cases and controls using linear mixed-effects models, with group (MCI vs control) as a fixed effect and household pair as a random effect.

Supplementary Table 4. Correlations between genus-level microbes and metabolites in the diagnosis of MCI.

Supplementary Table 5. Correlations between genus-level microbes and metabolites in the prognosis of MCI.

Supplementary Table 6. Classification of MCI Progression over 12 Months.

Supplementary Table 7. Sequence of primer used for 16S rRNA sequencing.
